# Transcriptomic Identification of Draxin-Responsive Targets During Cranial Neural Crest EMT

**DOI:** 10.3389/fphys.2021.624037

**Published:** 2021-02-03

**Authors:** Erica J. Hutchins, Michael L. Piacentino, Marianne E. Bronner

**Affiliations:** Division of Biology and Biological Engineering, California Institute of Technology, Pasadena, CA, United States

**Keywords:** Draxin, Wnt, neural crest, EMT, craniofacial development

## Abstract

Canonical Wnt signaling plays an essential role in proper craniofacial morphogenesis, at least partially due to regulation of various aspects of cranial neural crest development. In an effort to gain insight into the etiology of craniofacial abnormalities resulting from Wnt signaling and/or cranial neural crest dysfunction, we sought to identify Wnt-responsive targets during chick cranial neural crest development. To this end, we leveraged overexpression of a canonical Wnt antagonist, Draxin, in conjunction with RNA-sequencing of cranial neural crest cells that have just activated their epithelial–mesenchymal transition (EMT) program. Through differential expression analysis, gene list functional annotation, hybridization chain reaction (HCR), and quantitative reverse transcription polymerase chain reaction (RT-qPCR), we validated a novel downstream target of canonical Wnt signaling in cranial neural crest – *RHOB* – and identified possible signaling pathway crosstalk underlying cranial neural crest migration. The results reveal novel putative targets of canonical Wnt signaling during cranial neural crest EMT and highlight important intersections across signaling pathways involved in craniofacial development.

## Introduction

The neural crest is a multipotent stem cell population in the vertebrate embryo that undergoes coordinated induction, specification, and epithelial–mesenchymal transition (EMT) events to migrate and ultimately differentiate into a wide range of cell types. The migratory pathways and derivatives formed by the neural crest are regionalized according to their axial level of origin, such that cells from a given axial level give rise to a characteristic array of progeny and follow distinct pathways from those arising at other axial levels (Gandhi and Bronner, 2018). The most anterior “cranial” neural crest population underlies much of the development of the face (Cordero et al., 2011), and is the only neural crest population *in vivo* with the ability to differentiate into facial skeleton, contributing to the upper and lower jaw, and bones of the neck (Noden, 1975; Le Douarin, 1982; Simoes-Costa and Bronner, 2015). Importantly, perturbation of various stages of cranial neural crest development results in a myriad of craniofacial malformations (Vega-Lopez et al., 2018).

Many facets of cranial neural crest development are regulated by Wnt signaling (Wu et al., 2003; Yanfeng et al., 2003; Steventon et al., 2009; Milet and Monsoro-Burq, 2012; Simoes-Costa et al., 2015; Rabadán et al., 2016; Hutchins and Bronner, 2018, 2019; Gandhi et al., 2020). Furthermore, Wnt signaling is critical for normal facial patterning; mutations in Wnt pathway components or dysregulation of canonical Wnt signaling output result in defects in craniofacial morphogenesis (Huelsken et al., 2000; Chiquet et al., 2008; Reid et al., 2011; He and Chen, 2012; Kurosaka et al., 2014). Thus, identification of canonical Wnt targets during cranial neural crest development would greatly enhance understanding the etiology of craniofacial abnormalities resulting from Wnt signaling or cranial neural crest dysfunction.

Here, we took advantage of a canonical Wnt signaling inhibitor, Draxin, to identify Wnt-responsive targets during chick cranial neural crest development. As Draxin overexpression impedes cranial neural crest EMT in a β-catenin-dependent mechanism (Hutchins and Bronner, 2018, 2019), here we utilize Draxin overexpression together with RNA-sequencing (RNA-seq) on sorted populations of cranial neural crest cells to identify novel downstream targets of canonical Wnt signaling during cranial neural crest EMT.

## Materials and Methods

### Embryo Electroporation and Expression Constructs

Electroporations were performed at Hamburger-Hamilton stage HH4 (Hamburger and Hamilton, 1951), using commercially available fertile chicken (*Gallus gallus*) eggs (Sunstate Ranch, Sylmar, CA, United States), as previously described (Hutchins and Bronner, 2018). The cranial neural crest-specific enhancer NC1.1m3:GFP (Simoes-Costa et al., 2012), Draxin overexpression (Hutchins and Bronner, 2018), BRE::GFP BMP reporter (Le Dreau et al., 2012), NC1-Δ90βcat canonical Wnt signaling activation (Hutchins and Bronner, 2018), and control expression (Betancur et al., 2010b) constructs were described previously.

### Tissue Dissociation and FACS

Following electroporation, embryos were incubated at 37°C until HH9+. We then dissected embryonic heads anterior to the otic vesicle in Ringer’s solution, washed tissue with sterile PBS (Corning cellgro #21-031-CV), then incubated tissue in Accumax (Innovative Cell Technologies, Inc. #AM-105) at 37°C for 15 min, with trituration every 5 min. Following dissociation, cells were washed with Hanks’ Balanced Salt Solution (Thermo Fisher Scientific #88284), filtered through a 20 μM nylon net mesh filter (Millipore Product #NY2004700), and resuspended in Hanks’ supplemented with 0.25% bovine serum albumin and 5% RQ1 DNase (Promega #M6101). GFP + cells were then collected using fluorescence activated cell sorting (FACS) at the Caltech Flow Cytometry Cell Sorting Facility.

### Library Preparation and Sequencing

We used 1500 GFP+ cranial neural crest cells per replicate to prepare libraries. cDNA libraries were prepared using the Takara Bio SMART-Seq v4 Ultra Low Input cDNA kit, according to manufacturer instructions. RNA-Seq was performed at the Caltech Millard and Muriel Jacobs Genetics and Genomics Laboratory at 35 million reads on two biological replicates for both the control cranial and Draxin overexpression cranial neural crest cells. Sequencing libraries were built according to Illumina Standard Protocols and SR50 sequencing was performed in a HiSeq Illumina machine by the Caltech Millard and Muriel Jacobs Genetics and Genomics Laboratory. Sequence reads were aligned to the *G. gallus* genome (*galgal6*) with Bowtie2 (Langmead and Salzberg, 2012), transcript counts were calculated with HTSeq-Count (Anders et al., 2015), and differential expression analysis was performed with DESeq2 (Love et al., 2014). Gene lists were analyzed for functional annotation using PANTHER (Mi et al., 2019) and DAVID (Huang da et al., 2009a,b).

### Hybridization Chain Reaction

Embryos to be processed for hybridization chain reaction (HCR) were fixed in 4% paraformaldehyde 1 h at room temperature, then dehydrated in graded methanol washes and stored at least one overnight at −20°C. HCR was performed as previously described (Gandhi et al., 2020), with custom probes designed and ordered through Molecular Technologies.

### Image Acquisition and Analysis

Confocal images were acquired with an upright Zeiss LSM 880 at the Caltech Biological Imaging Facility. Images were minimally processed for brightness/contrast and pseudocolored using Fiji (Schindelin et al., 2012) and Adobe Photoshop 2020. Relative fluorescence intensity was determined in Fiji. For each whole mount image, the line tool was used to draw an ROI surrounding the area of neural crest indicated by positive HCR fluorescence for neural crest marker *TFAP2β*. Integrated density measurements were quantified for ROIs on the control electroporated (left) and experimental electroporated (right) sides from the same embryo. Relative fluorescence intensity was then calculated by dividing the integrated density measurements for the experimental versus the control side of the same embryo. Statistical analyses were performed using Prism (8; GraphPad Software). *P* values are defined in the text, and significance was established with *P* < 0.05. *P* values were calculated for embryos using one-tailed paired *t*-tests with integrated density measurements for control versus experimental sides, and for qRT-PCR using two-tailed one sample *t*-tests for ΔΔC_*T*_ values.

### Quantitative Reverse Transcription PCR (RT-qPCR)

RNA was extracted from sorted cells (Draxin overexpression) and dissected HH9+ embryonic half heads (NC1-Δ90βcat canonical Wnt signaling activation) using the RNAqueous-Micro Total RNA Isolation Kit (Invitrogen), according to manufacturer instructions. Following RNA isolation in elution buffer, cDNA was reverse transcribed using the SuperScriptIII First-Strand Synthesis System (Invitrogen) with oligo-dT priming. Quantitative PCR (qPCR) was performed using gene-specific primers with FastStart Universal SYBR Green Master Mix with Rox (Roche) and cDNA (diluted 1:10) on a QuantStudio 3 Real-Time PCR System (Applied Biosystems) in triplicate. We determined ΔC_*T*_ with normalization against 18S ribosomal RNA (ΔC_*T*_ = Target C_*T*_ − 18S C_*T*_) for *Draxin*, *SNAI2*, and *RHOB* for samples, then calculated ΔΔC_*T*_ values (ΔΔC_*T*_ = Average Control ΔC_*T*_ − Perturbation ΔC_*T*_) for each target and replicate. The gene-specific primers used for qPCR were: Draxin-F 5′-CTACGCTGTTATGCCAAATTCC; Draxin-R 5′-GAATGATCCCTGCTCTCCATT; SNAI2-F 5′-GCA ACAAGACCTATTCCACTTTC; SNAI2-R 5′-GTACTTG CAGCTGAACGATTTC; RHOB-F 5′-CGTGATCCTCATGT GCTTCT; RHOB-R 5′-TGCGCAGGTCTTTCTTGT; 18S-F 5′-CCATGATTAAGAGGGACGGC; 18S-R 5′-TGGCAAA TGCTTTCGCTTT.

## Results

### Identification of Draxin-Responsive Genes in Migrating Cranial Neural Crest

**FIGURE 1:**
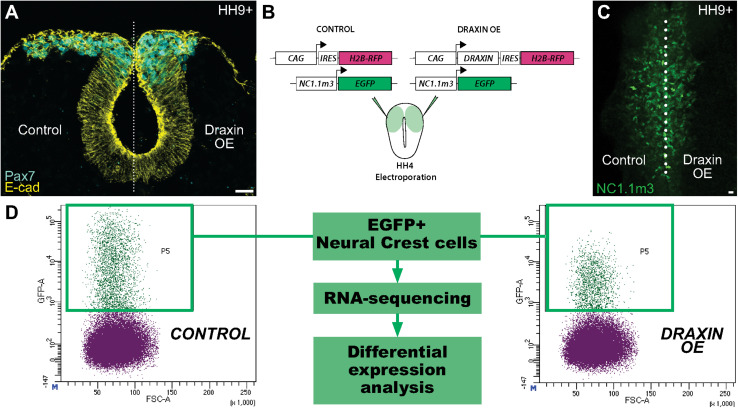
Experimental design for identification of Draxin-responsive targets in cranial neural crest EMT. **(A)** Cross-section through embryo electroporated with a Draxin overexpression construct on the right side of the embryo. Immunostaining for E-cadherin (E-cad) and the neural crest marker Pax7 at HH9+ highlights the deleterious effects of Draxin overexpression on cranial neural crest EMT and migration away from the midline (dotted line), compared to the contralateral control side. **(B–D)** Experimental design to isolate cranial neural crest cells with or without Draxin overexpression. Gastrula stage chick embryos were co-electroporated with a neural crest-specific enhancer driving EGFP expression in cranial neural crest cells (NC1.1m3) and either a Draxin overexpression or control construct **(B)**. NC1.1m3 enhancer expression revealed EGFP + cranial neural crest cells were responsive to Draxin overexpression and exhibited EMT defects **(C)**. Fluorescence activated cell sorting (FACS) was used to isolate EGFP+ cranial neural crest cells with and without Draxin overexpression that were subsequently processed for RNA-sequencing and differential expression analysis **(D)**. Scale bar, 20 μm. HH, Hamburger-Hamilton stage; OE, overexpression; IRES, internal ribosomal entry site.

We have previously shown that the secreted protein Draxin functions as a potent inhibitor of cranial neural crest cell migration during EMT (Figure 1A; Hutchins and Bronner, 2018, 2019). Its effects on neural crest are elicited extracellularly via β-catenin-dependent Wnt signaling inhibition, precisely at the early stages of cranial neural crest EMT at HH9+ (Hutchins and Bronner, 2018). To parse the cranial-specific targets of Draxin underlying its effect on neural crest EMT and uncover potential novel targets of canonical Wnt signaling, we performed RNA-sequencing (RNA-seq) on sorted chick cranial neural crest cells, with and without Draxin-mediated Wnt inhibition. To this end, we co-electroporated the FoxD3 NC1.1m3 enhancer, which drives GFP expression specifically in the cranial neural crest population (Simoes-Costa et al., 2012), with either a Draxin overexpression construct containing an internal ribosomal entry site (IRES) driving H2B-RFP (Hutchins and Bronner, 2018) or the same construct without the Draxin coding region as a control (Figure 1B). Embryos were subsequently developed to the onset of cranial neural crest EMT (HH9+), by which point Draxin-mediated effects on EMT are evident (Figure 1C; Hutchins and Bronner, 2018, 2019), to identify EMT-related genes sensitive to canonical Wnt inhibition. From heads dissected anterior to the otic vesicle, we isolated 1500 GFP+ cranial neural crest cells per replicate by FACS, then performed cDNA library preparation and sequencing (Figure 1D).

**FIGURE 2:**
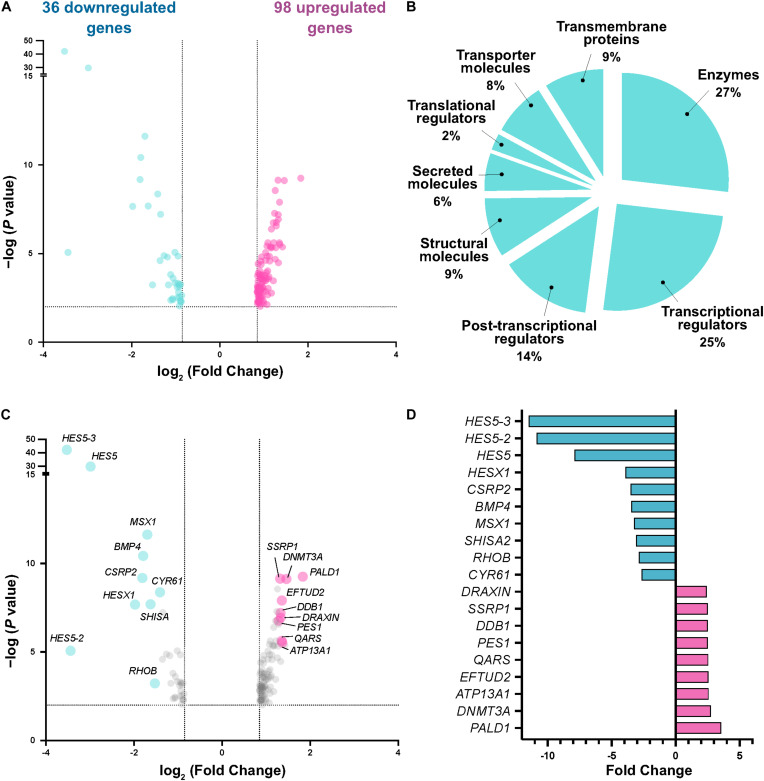
Transcriptome analysis reveals differentially expressed genes responsive to Draxin-mediated canonical Wnt inhibition. **(A)** Volcano plot following differential expression analysis and filtering. Dotted lines indicate significance and fold change cutoffs for downstream analysis. **(B)** Molecular functions for the 134 differentially expressed genes. **(C,D)** Most enriched and depleted genes in cranial neural crest with Draxin overexpression.

Differential expression analysis initially revealed 284 differentially expressed genes with ≥1.8 fold change and FDR < 0.01. For subsequent functional analysis, we filtered the gene lists to exclude lowly expressed genes (average normalized count values < 1000), resulting in a filtered list of 134 differentially expressed genes (36 downregulated, 98 upregulated) (Figure 2A). Using PANTHER analysis (Mi et al., 2019) to probe molecular functions of these gene targets, we observed enrichment of factors highly associated with transcriptional regulation, enzymatic reactions (including kinases) and secreted proteins indicative of targets associated with intracellular signaling pathways, and structural molecules (such as cytoskeletal and extracellular matrix proteins) indicative of cell migration-associated targets (Figure 2B). Among the most highly changed genes, we found significant enrichment of *Draxin*, as expected due to its experimental overexpression. Interestingly, we also detected significant downregulation of the Notch pathway effector *HES5* (and related genes), and *BMP4* (as well as its downstream target *MSX1*) (Figures 2C,D), suggesting potential signaling pathway crosstalk between Draxin, canonical Wnt signaling, and other pathways with critical roles in neural crest development. Given that Draxin has been shown to intersect with additional signaling pathways in other contexts (Ahmed et al., 2011; Hossain et al., 2013; Meli et al., 2015), further studies are needed to parse direct and indirect effects relevant to craniofacial morphogenesis and neural crest EMT.

### Biological Pathway Analysis of Draxin-Responsive Genes in Cranial Neural Crest

**FIGURE 3:**
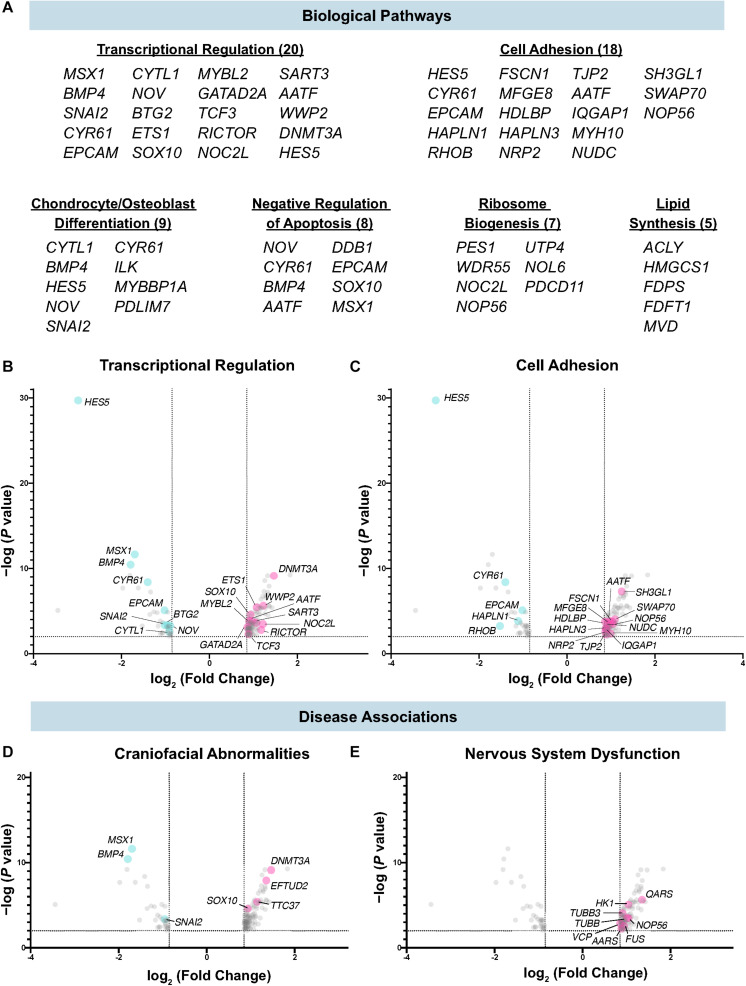
Biological pathways and diseases associated with Draxin-responsive transcriptome dataset. **(A–C)** Biological pathway analysis identified enrichment of targets associated with transcriptional regulation, cell adhesion, chondrocyte/osteoblast differentiation, negative regulation of apoptosis, ribosome biogenesis, and lipid synthesis. **(D,E)** Functional annotation identified genes highly correlated with craniofacial abnormalities and nervous system dysfunction.

To better understand the molecular processes in which Draxin, and by extension canonical Wnt signaling, function during cranial neural crest EMT, we performed functional annotation for the dataset using the Database for Annotation, Visualization and Integrated Discovery (DAVID) (Huang da et al., 2009a,b). Consistent with established roles of canonical Wnt signaling and Draxin-mediated inhibition during cranial neural crest EMT, we observed enrichment of genes associated with transcriptional regulation, cell adhesion, and lipid synthesis, which we have recently shown is important for cell signaling during cranial neural crest EMT (Piacentino et al., 2020). In addition, we found numerous genes associated with bone/cartilage formation (e.g., *CYTL1*, *ILK*, *NOV*), a critical function of cranial neural crest, and genes involved in ribosome biogenesis (e.g., *NOP56*, *PES1*, *NOC2L*), which has implications for craniofacial development (Ross and Zarbalis, 2014) (Figure 3A).

Among the targets associated with transcriptional regulation, we detected significant downregulation of *SNAI2*, which has been shown to be a direct target of canonical Wnt signaling, and subsequently Draxin (LaBonne and Bronner-Fraser, 1998; Monsoro-Burq et al., 2005; Wu et al., 2005; Hutchins and Bronner, 2018) (Figure 3B). In addition, we observed significant downregulation of genes that mediate cell adhesion and EMT (Figure 3C), including *RHOB*, shown to be required for neural crest delamination in the trunk (Liu and Jessell, 1998), and *EPCAM*, which has been shown to participate in cancer cell EMT (Wang et al., 2018). We also observed significant correlations for disease-associated genes, including those involved in craniofacial (Figure 3D) and nervous system dysfunction (Figure 3E). This was expected given the role of the cranial neural crest in craniofacial development, and established roles for Draxin in nervous system development (Islam et al., 2009; Tawarayama et al., 2018).

### Functional Validation of a Novel Immediate Early Canonical Wnt Target

**FIGURE 4:**
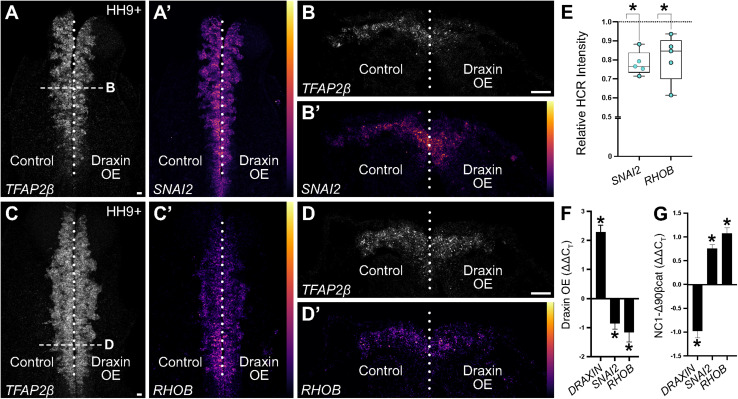
Hybridization chain reaction (HCR) validation of identified neural crest EMT genes. **(A–D)** Representative images of quantitative HCR for neural crest marker *TFAP2β* and targets *SNAI2* and *RHOB* in embryos bilaterally electroporated with a control (left) or Draxin overexpression (right) construct. *SNAI2* and *RHOB* are pseudocolored based on fluorescence intensities as indicated by color scale in panels **(A′–D′)**. **(E)** Integrated density measurements revealed significant downregulation of *SNAI2* and *RHOB* (78.0 ± 2.8% of the control side for *SNAI2* and 81.0 ± 5.5% of the control side for *RHOB*; *P* ≤ 0.01, one-tailed paired *t*-test) with Draxin overexpression. Scale bar, 20 μm. **(F)** ΔΔC_*T*_ values from quantitative reverse transcription PCR (RT-qPCR) for *DRAXIN*, *SNAI2*, and *RHOB* (normalized against *18S*, comparing control versus overexpression cells) from HH9+ sorted cranial neural crest cells co-electroporated with NC1.1m3 fluorescent GFP reporter with or without Draxin overexpression (DraxinOE). As expected, *DRAXIN* was significantly upregulated whereas *SNAI2* and *RHOB* were significantly downregulated in DraxinOE cranial neural crest cells. *P < 0.05, two-tailed one sample *t*-test. **(G)** ΔΔC_*T*_ values from quantitative reverse transcription PCR (RT-qPCR) for *DRAXIN*, *SNAI2*, and *RHOB* (normalized against *18S*, comparing control versus Wnt-activated cells) from dissected HH9+ cranial tissue from embryos co-electroporated with NC1.1m3 fluorescent RFP reporter with or without stabilized β-catenin expression (NC1-Δ90βcat). In contrast to Wnt inhibition via DraxinOE **(F)**, *SNAI2* and *RHOB* were significantly upregulated with Wnt activation via NC1-Δ90βcat, whereas *DRAXIN* was significantly downregulated. *P ≤ 0.02, two-tailed one sample *t*-test.

To validate Draxin-responsive targets from our dataset, we performed quantitative HCR on embryos bilaterally electroporated with the Draxin overexpression construct on the right side of embryos and the control construct on the left side (as in Figure 1B). To establish the area of neural crest migration from which to measure target fluorescence intensities, we visualized expression of *TFAP2β*, a neural crest marker and non-target of Draxin. We focused on early HH9 + embryos, corresponding to the beginning of cranial neural crest EMT and initial stages of migration, in order to probe immediate early gene changes. As a result, modest defects were evident in the distance cranial neural crest cells migrated away from the midline (Figures 4A–D), consistent with a Draxin overexpression phenotype, albeit to a lesser extent than later stage HH9+ embryos in which migration has progressed more laterally (Figures 1A,C; Hutchins and Bronner, 2018, 2019). We measured *SNAI2* and *RHOB* fluorescence intensities for Draxin overexpression versus control sides of individual embryos, and found significant downregulation of gene expression (Figure 4E; 78.0 ± 2.8% of the control side for *SNAI2* and 81.0 ± 5.5% of the control side for *RHOB*; *P* ≤ 0.01, one-tailed paired *t*-test), consistent with predicted trends based on our transcriptomic analyses. This is consistent with our previously published work indicating that Draxin acts upstream of Snail2 protein expression (Hutchins and Bronner, 2018). We further validated the effects of Draxin overexpression on *SNAI2* and *RHOB* using quantitative reverse transcription PCR (RT-qPCR) with sorted cells collected alongside sequenced cells from Figure 1; consistent with the HCR data (Figures 4A–E), we detected significant downregulation of both *SNAI2* and *RHOB* with Draxin overexpression (Figure 4F).

Given that *RHOB* has been previously shown to be a BMP-responsive target (Liu and Jessell, 1998) and insensitive to Wnt signaling (Taneyhill and Bronner-Fraser, 2005) in trunk neural crest, we next sought to determine whether the reduction in *RHOB* we observed in cranial neural crest was due to direct effects from Wnt signaling, or indirect effects through downregulation of BMP. We have previously shown that canonical Wnt signaling is active in cranial neural crest at the onset of EMT using a fluorescent reporter (Hutchins and Bronner, 2018), while BMP signaling is active in the presumptive cranial neural crest at earlier stages during their induction (Piacentino and Bronner, 2018); here we employed a similar approach to investigate the timing of BMP signaling activation in cranial neural crest at the onset of EMT. Electroporation of a fluorescent BMP reporter (BRE::GFP) revealed a lack of active BMP signaling in cranial neural crest cells that have undergone EMT and commenced migration at HH9+ (Supplementary Figure 1). Thus, it is unlikely that the reduction in *RHOB* we observed was due to suppressive effects on BMP signaling by Draxin. To more fully examine whether *RHOB* downregulation was due to direct effects from Wnt signaling, we performed RT-qPCR on embryos with and without canonical Wnt signaling activation during cranial neural crest EMT. Here, we specifically activated canonical Wnt signaling in specified cranial neural crest by driving expression of a stabilized form of β-catenin under the control of a neural crest-specific enhancer (NC1-Δ90βcat). Importantly, we observed upregulation of *SNAI2*, an established direct target of canonical Wnt signaling (LaBonne and Bronner-Fraser, 1998; Monsoro-Burq et al., 2005; Wu et al., 2005), as well as upregulation of *RHOB*, suggesting a direct link with Wnt signaling. Interestingly, we also observed concomitant downregulation of endogenous *DRAXIN*, suggesting the possibility of a negative feedback loop with respect to *DRAXIN* expression (Figure 4G).

Taken together, our data identify a novel target of Draxin and canonical Wnt signaling during cranial neural crest EMT (*RHOB*), and suggest that *Draxin* downregulation, and subsequent activation of Wnt signaling, is essential for crosstalk and feedback of signaling pathways that alter cranial neural crest transcriptional activation, and ultimately EMT.

## Discussion

Using transcriptome profiling of Draxin-responsive targets, we identified likely gene targets of canonical Wnt signaling during cranial neural crest EMT. Consistent with our previously published work examining protein expression (Hutchins and Bronner, 2018), we verified transcript downregulation of canonical Wnt target *SNAI2* in response to Draxin overexpression. Furthermore, we also identified and validated a novel target – *RHOB*. RhoB is BMP-responsive in trunk neural crest and is necessary for delamination (Liu and Jessell, 1998); its misexpression has been associated with defects in laminin organization within the basement membrane (Perez-Alcala et al., 2004). Interestingly, we have previously demonstrated that Draxin, via regulation of canonical Wnt signaling, also is involved in regulating laminin organization and remodeling of the basement membrane during cranial neural crest development (Hutchins and Bronner, 2019). Furthermore, we also observed downregulation of the BMP ligand *BMP4*, suggesting that BMP signaling may act downstream of Wnt signaling during or immediately after cranial neural crest EMT. In trunk, it has been shown that neural crest delamination is regulated by BMP, and that canonical Wnt signaling is controlled by BMP signaling through BMP-responsive expression of the *Wnt1* ligand (Burstyn-Cohen et al., 2004). This is particularly interesting in light of our observations from a GFP reporter construct that BMP signaling is inactive in cranial neural crest at the onset of EMT (Supplementary Figure 1). Interestingly, Draxin has also been shown to inhibit neural crest migration in the trunk (Su et al., 2009; Zhang et al., 2017). Thus, whether *RHOB* expression is differentially regulated based on axial level (i.e., in response to BMP signaling in trunk neural crest versus in response to Wnt signaling in cranial neural crest) or based on signaling pathway crosstalk (which may also be dependent on axial level) remains to be explored.

In searching our datasets for neural crest-specific factors, we also noted modest upregulation of *SOX10* and *ETS1*, genes associated with neural crest EMT (Tahtakran and Selleck, 2003; Theveneau et al., 2007; Simoes-Costa and Bronner, 2015), which seemed contradictory to the antagonistic role of Draxin in modulating cranial neural crest EMT. *ETS1* expression is restricted to the cranial population of neural crest and is itself activated via cMYB (Betancur et al., 2010a); together with Sox9, Ets1 and cMYB function as activating gene regulatory inputs into a *SOX10E2* enhancer (Betancur et al., 2010b), regulating *SOX10* expression in cranial neural crest. Interestingly, in other contexts, canonical Wnt signaling has been shown to trigger degradation of cMYB protein (Kanei-Ishii et al., 2004); given that *Draxin* is endogenously expressed at HH9, it is possible that its normal inhibitory effects on canonical Wnt signaling may be necessary to reduce degradation of cMYB to activate endogenous levels of *ETS1* and *SOX10*, which initiate expression prior to the onset of EMT. Thus, we postulate here that during early cranial neural crest migration, excess cMYB protein is stabilized via exogenous Draxin-mediated inhibition of canonical Wnt signaling; as a result, this may trigger upregulation of *ETS1* and *SOX10* gene expression. However, despite upregulation of factors positively associated with EMT, downregulation of *SNAI2* alone is sufficient to impede cranial neural crest migration (Nieto et al., 1994; Hutchins and Bronner, 2019).

Taken together, our data identify novel targets of canonical Wnt signaling during cranial neural crest EMT, and highlight potential avenues of intersection for signaling pathways involved in craniofacial development. The results raise the intriguing possibility that the sequence and magnitude of signaling and gene expression crosstalk during cranial neural crest development may help precisely regulate craniofacial morphogenesis.

## Data Availability Statement

The datasets presented in this study can be found in online repositories. The names of the repository/repositories and accession number(s) can be found below: https://www.ncbi.nlm.nih.gov/bioproject/PRJNA673315.

## Ethics Statement

Ethical review and approval was not required for the animal study because our study uses chicken embryos at E1-2. These are not considered vertebrate embryos until E10, and thus we do not require ethics committee approvals or protocols, as they are not considered vertebrates at the stages we work.

## Author Contributions

EH, MP, and MB conceived the project and conducted the experimental design and data interpretation. EH and MP performed the cell dissociations, library preparations, and RNA-seq analyses. EH performed the functional annotation, hybridization chain reaction experiments, imaging, quantitation, and statistical analyses. EH and MB wrote the manuscript with editing by MP. All authors contributed to the article and approved the submitted version.

## Conflict of Interest

The authors declare that the research was conducted in the absence of any commercial or financial relationships that could be construed as a potential conflict of interest.

## Supplementary Material

Supplementary Figure 1 BMP-sensitive reporter expression is absent from delaminating and migratory cranial neural crest during EMT. **(A)** Schematic of cross section of cranial region at HH9+ examined in panel **(B)**. Locations of premigratory (pNC) and migratory crest cells (mNC) are indicated. **(B)** Following electroporation of a BMP fluorescent reporter construct (BRE:GFP), cross sections stained for a neural crest marker (Snail2, magenta) and membrane label (WGA, white) revealed BMP reporter activity (green) in pNC (white dotted line), but not in delaminating or mNC (yellow dotted line). WGA, wheat germ agglutinin; pNC, premigratory neural crest; mNC, migratory neural crest. Scale bar, 10 μm.
