# Supplementary figures and images for: Transcriptomic Identification of Draxin-Responsive Targets During Cranial Neural Crest EMT
Source: Front Physiol. 2021 Feb 3;12:624037. doi: 10.3389/fphys.2021.624037 (PMC7886793; doi:10.3389/fphys.2021.624037)

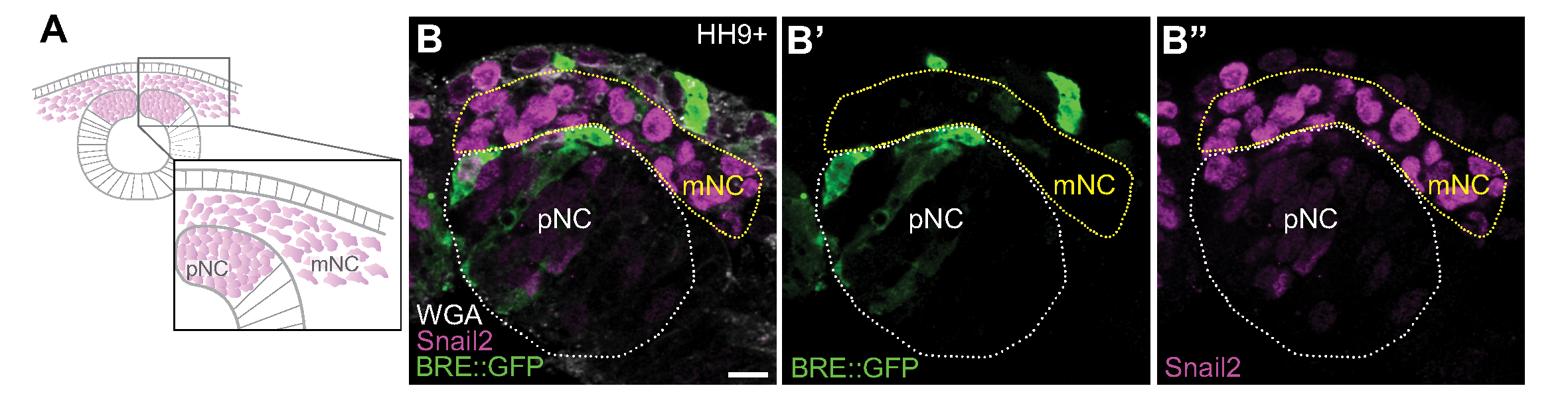

Supplement: Supplementary Figure 1 — BMP-sensitive reporter expression is absent from delaminating and migratory cranial neural crest during EMT. (A) Schematic of cross section of cranial region at HH9+ examined in panel (B). Locations of premigratory (pNC) and migratory crest cells (mNC) are indicated. (B) Following electroporation of a BMP fluorescent reporter construct (BRE:GFP), cross sections stained for a neural crest marker (Snail2, magenta) and membrane label (WGA, white) revealed BMP reporter activity (green) in pNC (white dotted line), but not in delaminating or mNC (yellow dotted line). WGA, wheat germ agglutinin; pNC, premigratory neural crest; mNC, migratory neural crest. Scale bar, 10 μm. [file Image_1.TIF]
